# Personality masks of mothers of young children: measure development and parenting behavior impact

**DOI:** 10.3389/fpsyg.2025.1674406

**Published:** 2025-11-19

**Authors:** Ruiqian Li, Siyao Yan, Senwei Fang, Jiaying Xu, Chenyu Sun, Tong Shen

**Affiliations:** Department of Early Childhood Education, School of Education and Psychology, Shaoxing University, Shaoxing, China

**Keywords:** mothers, personality masks, parenting behavior, child development, family wellbeing

## Abstract

This study developed and validated an instrument to assess the personality masks of mothers of young children, addressing a gap in the developmental psychology. Using a mixed-methods approach grounded in Carl Jung’s theory of persona, we collected data from 486 mothers in two-parent families in China’s Z region. The research involved three phases: scale development via open-ended questionnaires, which identified a three-dimensional structure of the maternal personality mask (Collective Ideals, Personal Ideals, and Physical/Mental Qualities); confirmatory factor analysis, which validated the model with excellent fit indices; and an implicit memory experiment, which explored the unconscious expression of these dimensions. Results showed significant correlations between the mask dimensions and various parenting behaviors, accounting for notable variance in parenting outcomes. These findings suggest that the personality mask is a valuable construct for understanding maternal behavior, and supporting balanced psychological adaptation in mothers may improve parenting effectiveness. The study provides a practical tool for future research and intervention, while also highlighting the need for further studies with greater cultural diversity and ecological validity.

## 1 Introduction

Early childhood development has profound implications for subsequent physical and mental growth (Shonkoff and Phillips, 2000). As primary caregivers, mothers’ parenting behaviors exert a significant influence on children’s social adjustment, emotional regulation, and cognitive development (Tamis-LeMonda et al., 2004; Thompson, 2008), with personality traits acting as the core factor shaping these parenting practices (Bornstein, 2019). Jung proposed the concept of the “persona”—a term widely adopted in psychology to refer to dynamic expressive structures shaped by societal expectations, which differ from an individual’s intrinsic traits (Jung, 2009, 2011). Subsequent scholars have expanded this framework: Guo (2012) and Samuels (2015) described the persona as a composite representation that integrates cultural norms, group consensus, and individual needs, while Jacobi (1976), Fan (2008), and Markus and Kitayama (1991) emphasized that cultural backgrounds influence its constituent dimensions.

Regarding preschool mothers, Belsky (1984) and Fan (2008) proposed that their persona constitutes a dynamic combination of traits actively constructed to align with social norms and family responsibilities, representing a dynamic equilibrium between personal characteristics, family environment, and sociocultural influences (Stern, 1995). Mothers adjust their behaviors according to “good mother” expectations (Hays, 1996), integrate self-growth needs (Brockington, 2004; Cash and Pruzinsky, 1990), and maintain stable physical and mental states as the foundation for consistent expression (Field, 2011; Goodman and Gotlib, 1999). This ultimately forms a three-dimensional construct encompassing collective ideals, personal aspirations, and physical-mental wellbeing—one that is distinct from intrinsic personality traits and passive social roles.

At the operational level, the evaluation framework builds upon established paradigms: Fan (2008) generated initial items through open-ended questionnaires, while Lee et al. (2017) implemented a joint expert-group scoring method to identify high-acceptance items. Reliability and validity evaluations adhered to the standards set by Franke and Byrne (1995) and Steiger (1990), with structural validation achieved through Exploratory Factor Analysis (EFA) and Confirmatory Factor Analysis (CFA). Furthermore, Lee et al. (2017) introduced implicit memory experiments as a supplement, establishing an “explicit + implicit” measurement pathway. Compared to traditional personality models, maternal personality masks in early childhood better adapt to specific contexts (Deater-Deckard et al., 2006), more aligned with local cultural norms (Markus and Kitayama, 1991), capture unconscious tendencies (Bargh and Morsella, 2008), and directly correlate with parenting behaviors (Kim, 2011). These insights address gaps in research on the underlying psychological mechanisms (Bornstein, 2019; Leerkes et al., 2016; Yaffe, 2020) and provide evidence-based foundations for parenting interventions.

For mothers of young children, personality masks are traits and behaviors adapted to social and family roles (Belsky, 1984; Fan, 2008). Influenced by personal traits, family environment, and sociocultural background (Stern, 1995), mothers conform to societal expectations for acceptance (Hays, 1996). They may display assertiveness at work and tenderness at home while striving to meet personal demands for confidence and self-respect (Brockington, 2004; Cash and Pruzinsky, 1990). Physical and mental health significantly affect maternal behavior, with unwell mothers struggling to meet children’s needs (Field, 2011; Goodman and Gotlib, 1999).

Maternal parenting encompasses various approaches and behaviors that significantly impact children’s growth and overall development (Landry et al., 2001). Kim (2011) divided mothers’ parenting behaviors into five attitude categories: affectionate–rejection, autonomous–controlling, democratic–authoritarian, intentional indifference–overprotective, and consistency–inconsistency.

Affectionate parenting fosters proactive, sociable, creative, and emotionally stable children (Grolnick and Pomerantz, 2016; Zhang et al., 2020). Rejection involves hostility or neglect, harming children’s emotional wellbeing (Kim et al., 2013). Autonomous parenting supports independence, promoting responsibility (Deci and Ryan, 1985; Grolnick and Ryan, 1989). Controlling parenting induces fear and anxiety (Aunola and Nurmi, 2005). Democratic parenting encourages expression and self-control (Baumrind, 1991; Deci and Ryan, 2000). Consistent parenting provides clear boundaries and support, while inconsistent parenting fosters rebellion and low self-confidence (Piotrowski et al., 2013; Deci and Ryan, 2000).

Research on parenting by mothers of young children generally focuses on mothers’ parenting behaviors, while neglecting the underlying psychological factors (Bornstein, 2019; Leerkes et al., 2016; Yaffe, 2020). Mothers of young children may exhibit similar parenting behaviors, despite having different personalities and backgrounds, owing to the influence of the collective unconscious. To gain insight into maternal parenting behaviors, an evaluation tool to assess a mother’s collective unconscious and conscious level is needed. Thus, this study aims to develop an assessment tool for measuring mothers’ personality masks, and then use it in an implicit memory experiment to explore their relationship with maternal parenting behaviors.

This study includes three research questions: (1) What is the structure of the personality masks used by mothers of young children? (2) What are the manifestations of personality masks in mothers of young children? (3) What is the relationship between mothers’ personality masks and their parenting behaviors?

## 2 Materials and methods

### 2.1 Research design and framework

Research Design and Overall Framework: This study employs a sequential mixed-methods design to systematically investigate the structure of maternal persona among preschool children’s mothers and its relationship with parenting behaviors across three phases. Phase 1 involves developing an assessment tool for persona through open-ended questionnaires and factor analysis. Phase 2 validates the unconscious expression of persona using implicit memory experiments. Phase 3 examines the association between persona and parenting behaviors via correlation analysis. This design ensures coherence from theoretical construction to empirical verification, with findings from each phase complementing one another: Results of factor analysis directly inform the selection of experimental materials, while experimental data cross-validate outcomes of the questionnaire.

### 2.2 Participants

#### 2.2.1 Total sample

The primary participants of this study were mothers of preschool children in Region Z of China. All participants were from two-parent families, with their children aged 3–6 years and enrolled in local regular kindergartens. The total sample was recruited via a multi-stage process, covering kindergartens in Region Z. The final valid sample size was 486, with breakdowns as follows: 153 participants in the lexical construction phase, 233 in the factor analysis phase, and 100 in the implicit memory experiment phase. In terms of age distribution, the majority of participants were 26–35 years old (accounting for 53.6–56%), followed by those aged ≥36 years (27.5–32.7%) and ≤25 years (13.7–18.0%). All mothers had basic Chinese literacy skills and participated voluntarily in the study. The sample exhibited high homogeneity in regional and cultural backgrounds, with 82% being urban residents.

#### 2.2.2 Research question 1

Participants in this study were recruited in two sub-phases: (1) Vocabulary Construction Sub-phase: A total of 200 mothers were recruited from Kindergarten H in Region Z, with 153 valid questionnaires collected. Their age distribution was as follows: ≤25 years old (21 participants, 13.7%), 26–35 years old (82 participants, 53.6%), and ≥36 years old (50 participants, 32.7%). (2) Factor Analysis Sub-phase: A total of 233 mothers were recruited from Kindergarten J in Region Z. Their age distribution was: ≤25 years old (42 participants, 18.0%), 26–35 years old (127 participants, 54.5%), and ≥36 years old (64 participants, 27.5%). All participants were from two-parent families in Region Z. Data were collected via open-ended questionnaires and Likert scales, ensuring consistency in family structure, parenting stage, and cultural background across the sample.

#### 2.2.3 Research questions 2

One-hundred mothers of young children were recruited from Kindergarten X in Z District to participate in this experiment. The majority were aged 26–35 years (56%), followed by ≥36 years (28%) and ≤25 years (16%). The inclusion criteria for all participants are as follows: In terms of basic demographic characteristics, participants must be biological mothers, with their children strictly aged between 3 and 6 years old and enrolled in regular kindergartens. This criterion ensures all participants are in the same parenting stage, reducing variations in parenting behaviors caused by differences in children’s ages. Regarding family structure, participants must come from two-parent households (i.e., living with their spouse). This criterion controls for potential influences of family support systems, as single-parent families may face distinct parenting pressures and resource conditions. For geographical and cultural backgrounds, all participants must have resided in Region Z for at least 2 years to ensure consistency in cultural context and regional educational policies, minimizing the interference of cross-cultural differences on the expression of personality masks. In terms of language and cognitive abilities, participants must possess basic Chinese literacy skills to independently complete questionnaires and experimental tasks. Their cognitive adaptability was verified through screening items (e.g., understanding experimental instructions) during the pre-test phase.

This study implemented a systematic exclusion mechanism to control for confounding variables. The exclusion criteria are specified as follows: For special groups: Mothers whose children have diagnosed developmental disorders (e.g., autism, ADHD) are excluded, as their parenting behaviors may exhibit systematic differences; mothers with severe mental health issues (screened via the Symptom Checklist-90 [SCL-90], with those scoring > 160 excluded); and families that are single-parent, blended, or where grandparents primarily assume parenting responsibilities. For data quality control: Dynamic exclusion was applied during the data collection phase. Questionnaire responses were excluded if the response time was insufficient (< 5 min) or if there was patterned responding (e.g., selecting the same option for 10 consecutive questions). In the implicit memory experiment, data from participants with an error rate > 30% or abnormal reaction times (±3 standard deviations) were excluded.

Participants completed the computerized experiment in a quiet, controlled environment, with the E-Prime program used to record reaction times and J ratios. A randomized design was employed to control for order effects and practice effects. The characteristics of this sample were consistent with those of the overall study participants (two-parent families with children aged 3–6 years), but it was independent of the sample used for Research Question 1 to ensure the purity and validity of the experimental data.

#### 2.2.4 Research questions 3

The participants in this study were the same as those in research question 2, involving 100 mothers with an age distribution matching the previous cohort. Immediately after completing the implicit memory experiment, these participants completed a parenting behavior questionnaire developed by Kim (2011), which evaluates five dimensions including emotional rejection and autonomy-control.

### 2.3 Procedure

#### 2.3.1 Research questions 1

The study followed a systematic scale development process, implemented as follows: First, open-ended questionnaires were designed based on the theoretical framework to collect descriptions of “good mother” behaviors from 200 mothers; 102 initial statements were generated through text analysis. A review panel—comprising four experts and five mothers—was then formed to conduct dual-track independent reviews via the Delphi method (experts focused on theoretical dimension alignment, mothers on practical parenting scenarios). Only statements scoring 10–13 (≥75% expert agreement and ≥10 points from mothers) were included. This approach balanced external (expert-led) and internal (participant-centered) perspectives, thereby enhancing the content validity of the initial instrument. Additionally, item deletion in EFA strictly adhered to objective statistical criteria (e.g., factor loadings > 0.6, no significant cross-loadings) rather than subjective judgment, ensuring a robust and interpretable factor structure. Per the scoring criteria, 45 statements were retained. Subsequently, a 5-point Likert scale was administered to 233 mothers, and EFA was conducted using SPSS 25.0. Data suitability was confirmed via the KMO test (0.903) and Bartlett’s Test of Sphericity (*p* < 0.001), after which principal component analysis and varimax rotation were employed. Factors were extracted iteratively based on the criteria of eigenvalues > 1, factor loadings > 0.6, and cross-loading differences > 0.05. Finally, Confirmatory Factor Analysis (CFA) was performed using AMOS 24.0, with model fit evaluated through structural equation modeling. All analyses strictly followed psychometric standards. The entire process was approved by the Ethics Committee, and quality control measures—including dual-person data entry and random follow-up—were implemented.

#### 2.3.2 Research questions 2

Computerized Implicit Memory Experiment Protocol: Based on the factor analysis results of Research Question 1, 13 core statements were selected as experimental materials. All statements were standardized to 7 Chinese characters in length, with 2 randomly highlighted red characters. A total of 100 mothers were recruited to participate in the experiment, where stimuli were presented via standardized computer equipment and E-Prime software to control for environmental and technical variables. Following the implicit memory methodology by Lee et al. (2017), participants completed three phases programmed with E-Prime 3.0: ① Sentence Learning Phase: Participants performed a distractor task—judging whether the number of strokes of red Chinese characters exceeded 10 (by pressing the “J” or “F” key)—to unconsciously memorize the 13 target statements (controlling for visual attention and memory load). ②Memory Interference Phase: Counting random geometric shapes was used to further reduce explicit memory. ③Sentence Recognition Phase: Participants identified previously seen statements from a list of 13 target statements and 13 irrelevant distractor statements; only data from target statements were included in analysis (distractor statements were used to test false alarm rates and excluded). Thirteen distractor sentences (data not analyzed) were included to control for order effects. The software automatically recorded the J ratio (sentence recall accuracy) and reaction times in milliseconds. A pilot study with 6 mothers was first conducted to validate procedural feasibility, followed by a single-blind formal experiment conducted independently in quiet environments to ensure data standardization.

To effectively control the interference of potential confounding variables on experimental results, multiple measures were implemented in the design of the implicit memory experiment. First, the presentation order of all experimental materials was fully randomized via E-Prime software to eliminate order effects and fatigue effects caused by fixed sequences. Second, prior to the formal experiment, each participant completed practice trials with different materials to ensure they understood task requirements and became familiar with key-press responses—thereby reducing the contamination of reaction time data by learning curves. Additionally, a within-subjects design was adopted, where all target statements and distractor statements were balanced across experimental blocks. Finally, during the data cleaning phase, objective exclusion criteria were preset (e.g., error rate > 30% or reaction times outside ±3 standard deviations) to ensure only data from participants who maintained focus and demonstrated reliable task performance were included in the final analysis. These measures collectively ensured that observed differences in the J ratio and reaction times more accurately reflected the unconscious expression of personality masks, rather than being influenced by irrelevant variables such as experimental procedures or participants’states.

#### 2.3.3 Research questions 3

Quantitative research methodology: A quantitative correlational research method was adopted: After a cohort of 100 mothers completed the implicit memory experiment, they were immediately administered the Maternal Parenting Behavior Questionnaire developed by Kim (2011) — this was to ensure the synchrony and comparability of behavioral data and questionnaire data. The questionnaire comprises 50 items across five dimensions (Affection-Rejection, Autonomy-Control, Democracy-Authoritarian, Intentional Neglect-Overprotection, Consistency-Inconsistency) and uses a 5-point Likert scale. Researchers provided on-site guidance during questionnaire administration; the questionnaire was administered by the researchers on site and filled out anonymously. Data were statistically analyzed using SPSS 25.0: First, descriptive analysis was used to characterize the sample’s demographic features. Second, analysis of variance (ANOVA) was employed to examine differences in parenting behavior dimensions across groups classified by persona dimensions (grouped based on experimental J ratios). Finally, correlational analysis was used to calculate the strength of the association between persona traits and parenting behaviors. For all analyses, a significance threshold of *α* = 0.05 was set, and corresponding effect size indicators were reported.

### 2.4 Measures

#### 2.4.1 Research questions 1

The core research instrument was the self-developed Preliminary Screening Questionnaire for the Persona of Mothers of Preschoolers. Its development went through three phases: Firstly, an initial item pool was developed by designing open-ended questions based on Fan’s (2008) theoretical framework. Secondly, a dual-track evaluation was conducted by a review panel consisting of four psycho-educational experts and five mothers: experts focused on alignment with theoretical dimensions, while mothers assessed relevance to real-life scenarios. Thirdly, the 102 initial items underwent a dual review following the standardized procedure proposed by Lee et al. (2017), resulting in a finalized 45-item questionnaire (selection rate: 44.1%). A 5-point Likert scale was adopted, where 1 = “Not at all Consistent” and 5 = “Completely Consistent.” According to the expert rating data (Table 1): All selected items achieved an Item-Level Content Validity Index (I-CVI) of ≥0.75 (expert agreement rate≥ 75%), and the Scale-Level Content Validity Index (S-CVI/Ave) reached 0.89—exceeding the critical threshold of 0.70. Additionally, the average rating from the target group was 11.5 points (range: 10–13 points), which was significantly higher than the preset threshold of ≥10 points.

**Table 1:** Personality mask statement scoring results.

Statement	Expert				MYC	Score
	1	2	3	4		
1. Strong cultural literacy	2	0	2	2	5	11
2. High self-confidence and self-esteem	2	2	2	0	5	11
3. Excellent communication skills	2	2	2	2	5	13
4. Strong autonomy	0	2	2	2	5	11
5. Demonstrates tolerance toward others	2	2	2	2	4	12
6. Exceptional leadership skills	2	2	0	2	5	11
7. Ability to exercise self-restraint	2	2	2	2	3	11
8. Brave and resilient spirit	2	2	2	2	5	13
9. Displays empathy toward others	2	2	2	2	5	13
10. Content with one’s possessions and circumstances	0	2	2	2	4	10
11. Willingness to assist others	2	2	2	2	3	11
12. Maintains a positive and happy demeanor	0	2	2	2	5	11
13. Capable of self-reflection	2	2	2	2	4	12
14. Strong values	2	2	2	2	5	13
15. Honest and ethical approach toward work	2	2	2	2	5	13
16. Demonstrates strong willpower	2	2	2	2	4	12
17. Good rapport with others	2	2	0	2	4	10
18. Patient in dealing with people	2	2	2	2	4	12
19. Polite and courteous in interactions with others	2	2	2	2	5	13
20. Exceptional decision-making skills	2	2	2	2	3	11
21. Possesses self-protection awareness	2	2	2	2	5	13
22. Strong self-management skills	2	2	2	2	3	10
23. Demonstrates wisdom in interpersonal relationships	2	2	2	2	4	12
24. Strong innovation ability	2	2	2	2	5	13
25. Exceptional self-regulation ability	2	2	2	2	4	12
26. Maintains emotional stability	2	2	2	2	5	13
27. Strong understanding ability	2	2	2	2	4	12
28. Respects others’ values and beliefs	2	2	2	2	5	13
29. Excellent listener	2	2	2	2	4	12
30. Strong learning ability	2	2	2	2	5	13
31. Exceptional adaptability	2	2	2	2	5	13
32. Strong self-awareness	2	2	2	2	5	13
33. Displays a spirit of selfless dedication	2	2	2	2	3	11
34. Willingness to share good things with others	2	2	2	2	5	13
35. Maintains psychological balance	2	2	2	2	4	12
36. Possessing a far-sighted vision	2	2	2	2	3	11
37. Good physical and mental health	2	2	2	2	5	13
38. Demonstrates a spirit of hard work and endurance	2	2	2	2	4	12
39. Optimistic and positive attitude	2	2	2	2	5	13
40. Demonstrates humility and prudence in handling situations	2	2	2	2	3	11
41. Strong sense of family responsibility	2	2	2	2	5	13
42. Able to care for family members	2	2	2	2	5	13
43. Maintains family harmony	2	2	2	2	2	13
44. Strong sense of social responsibility	2	2	2	2	2	13
45. Kindness and love toward family members	2	2	2	2	3	11

MYC, mothers of young children.

These quantitative indicators conclusively demonstrate that the scale items have excellent content representativeness and face validity across the three dimensions of collective ideals, personal aspirations, and physical-mental qualities. The reliability and validity of the instrument were verified through exploratory factor analysis (KMO = 0.903) and confirmatory factor analysis (CFI = 0.945), meeting psychometric standards. All data were collected using uniformly developed paper-based questionnaires, which were completed under the guidance of on-site researchers.

#### 2.4.2 Research questions 2

A computerized implicit memory experiment system was adopted, implemented via programming in E-Prime 3.0 software based on the paradigm proposed by Lee et al. (2017). The experimental program consisted of three core modules: In the sentence learning phase, 7-character sentences with Chinese characters marked in red were presented, and participants were required to judge whether the number of strokes of the marked characters was >10. In the memory interference phase, random geometric sequences were displayed, and participants needed to count the number of shapes. In the sentence reproduction phase, participants were asked to identify the sentences they had previously learned. The experimental materials included 13 target sentences (derived from the factor analysis results of Research Question 1) and 13 distractor sentences. All sentences were uniformly 7 characters in length, with 2 characters randomly marked in red. Data collection involved the program automatically recording the J ratio (sentence reproduction accuracy rate) and reaction times in milliseconds. The experiment was conducted in a standardized computer room using computers of the same model.

#### 2.4.3 Research questions 3

The Maternal Parenting Behavior Questionnaire developed by Kim (2011) was used as a standardized measurement tool. This questionnaire consists of 50 items, covering five core dimensions: Affection-Rejection (10 items), Autonomy-Control (10 items), Democracy-Authority (10 items), Intentional Neglect-Overprotection (10 items), and Consistency-Inconsistency (10 items). A 5-point Likert scale was adopted, where 1 = “Strongly Disagree” and 5 = “Strongly Agree.” After cross-cultural adaptation, the Cronbach’s *α* coefficients of the Chinese version ranged from 0.805 to 0.845. The questionnaire administration was conducted synchronously with the implicit memory experiment: paper-based questionnaires were distributed immediately after the experiment, and researchers explained the filling requirements on-site before collecting the questionnaires on the spot.

### 2.5 Data analysis

#### 2.5.1 Research questions 1

To explore the structure of persona, this study employed a series of factor analysis methods for data processing. First, the collected questionnaire data were cleaned and preprocessed using SPSS 25.0 software, including missing value handling (using the series mean imputation method) and normality testing (Shapiro–Wilk test). Subsequently, exploratory factor analysis (EFA) was conducted: the suitability of the data was evaluated using the Kaiser-Meyer-Olkin (KMO) measure and Bartlett’s test of sphericity. Principal component analysis was employed to extract factors, the number of factors was determined based on the criterion that eigenvalues are >1, and varimax rotation was applied. Items with factor loadings >0.6 and cross-loading differences >0.05 were retained. Finally, confirmatory factor analysis (CFA) was performed: a structural equation model was constructed using AMOS 24.0 software, and parameters were estimated by the maximum likelihood method. Model fit indices included χ^2^/df, CFI, TLI, RMSEA, and SRMR, among others. Additionally, composite reliability (CR) and average variance extracted (AVE) were calculated to test reliability and validity.

#### 2.5.2 Research questions 2

For addressing the research question regarding the unconscious expression of persona, experimental data processing and statistical analysis methods were employed: First, raw data were collected via the E-Prime 3.0 program, including two key indicators: J ratio (sentence reproduction accuracy rate) and reaction time (in milliseconds). The data underwent cleaning and preprocessing, with trials excluded if they had an error rate exceeding 30% or a reaction time outside the range of ±3 standard deviations. SPSS 25.0 was used for descriptive statistics (calculating means and standard deviations) and inferential statistics. One-way analysis of variance (ANOVA) was applied to compare differences in J ratio and reaction time across different persona dimensions (collective ideals, personal ideals, and physical-mental qualities).

#### 2.5.3 Research questions 3

For the research question regarding the relationship between personal and parenting behaviors, this study employed analysis of variance (ANOVA) methods: First, SPSS 25.0 was used to conduct reliability analysis on the parenting behavior questionnaire data, calculating Cronbach’s *α* coefficients to ensure the internal consistency of the scale. Subsequently, one-way analysis of variance (ANOVA) was performed to compare score differences in each dimension of parenting behaviors across groups classified by different persona dimensions. Inter-group comparisons were conducted using the ANOVA module in SPSS, with effect sizes calculated.

A significance threshold of α = 0.05 was set for all analyses, and continuous variables were tested for normality using the Shapiro–Wilk test.

## 3 Results

### 3.1 Research questions 1

**Figure 1:**
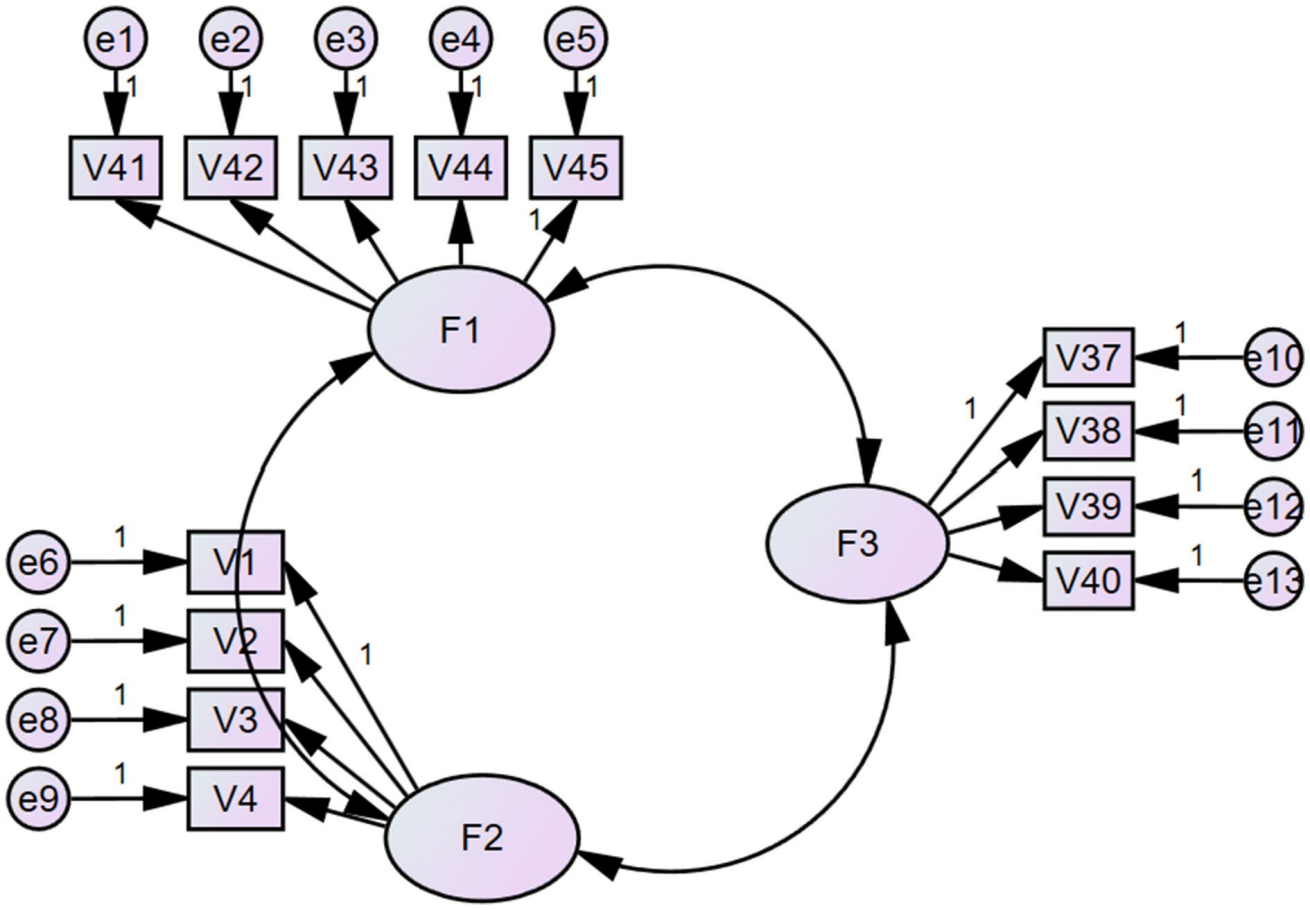
Results of confirmatory factor analysis.

An exploratory factor analysis (EFA) was performed analyzing relevant data using SPSS. The questionnaire had good reliability (Cronbach’s alpha = 0.936). The principal component method was used for factor extraction, and the maximum variance method was used for rotation. The following criteria were applied according to factor analysis theory: (1) Eigenvalue of factor > 1; (2) Meet the Scree Test; (3) Extracted factors should explain approximately 3% of total variation before rotation; (4) Each factor should contain more than three statements. Before performing the EFA, we used the Kaiser–Meyer–Olkin (KMO) and Bartlett tests to check whether the data were suitable for factor analysis. The KMO result of 0.903 (df = 0.990) indicates that the variables are strongly correlated, and factor analysis could be performed. Bartlett’s test result was significant at a *p* < 0.000 level, so the variables were correlated, and factor analysis was conducted. The EFA process and results were as follows: First, 10 factors were extracted from the 45 questions, with a cumulative variance of 60.86%. Owing to the large number of statements and factors extracted, determining which factors the statements belonged to was difficult; therefore, deleting the statement was necessary. The loading criteria were as follows: (1) factor loading < 0.6; (2) the load of statements on factors was similar, and their difference was <0.05; (3) the number of statements in a factor was ≤ 3. Statements 5, 6, 25, 35, 29, 13, 31, 30, 21, 9, 24, 18, 19, 10, 32, 15, 36, 16, 11, 20, with a load of less than 0.6, were deleted. The number of factors for statements 27, 17, 26, 28, 33, 34, 14, 12, 22, 23, 7, and 8 was less than three; therefore, these statements were deleted. The remaining 13 statements were divided into three factors. KMO = 0.812, Bartlett = 1069.065, and degrees of freedom (df) = 78, *p* < 0.001. The cumulative variance was 61.823%. Following Jacobi (1976), the present study named Factor 1 (v 41, v 42, v 43, v 44, v 45) as the collective ideals for mothers of young children; Factor 2 (v 1, v 2, v 3, v 4) as the individual ideals; and Factor 3 (v 37, v 38, v 39, v 40) as the physical and mental qualities. The details are listed in Table 2. Figure 1 shows the validation factor results.

**Table 2:** Exploratory factor analysis results.

Statements	Collective ideals	Individual ideals	Physical and mental qualities
v43	**0.828**	−0.008	0.024
v41	**0.775**	0.193	0.167
v42	**0.758**	−0.043	0.006
v45	**0.729**	0.223	0.096
v44	**0.718**	0.221	0.017
v2	0.180	**0.820**	0.278
v1	0.057	**0.811**	0.096
v3	0.131	**0.759**	0.165
v4	0.094	**0.753**	0.210
v39	0.069	0.215	**0.787**
v37	0.154	0.023	**0.772**
v38	−0.017	0.040	**0.739**
v40	0.039	0.134	**0.704**
Total	4.070	31.307	31.307
Percentage of variance	2.204	16.950	48.258
Cumulative %	1.763	13.565	61.823

v43 = Strong sense of family responsibility; v41 = Able to care for family members; v42 = Maintains family harmony; v45 = Strong sense of social responsibility; v44 = Kindness and love toward family members; v2 = Strong cultural literacy; v1 = High self-confidence and self-esteem; v3 = Excellent communication skills; v4 = Strong autonomy; v39 = Good physical and mental health; v37 = Demonstrates a spirit of hard work and endurance; v38 = Optimistic and positive attitude; v40 = Demonstrates humility and prudence in handling situations. The values in the bolded sections indicate that the factor loadings are greater than 0.6.

*Validation factor analysis results.* A confirmatory factor analysis was conducted to verify the fit between the various factors. The analysis of a moment structures (AMOS) fit test showed that the minimum discrepancy function by degrees of freedom divided (CMIN/*df*) = 1.906 (ideal range = 1–3); the root mean square error of approximation (RMSEA) = 0.062 (ideal range < 0 08); the root mean square residual (RMR) = 0.053 (ideal range < 0. 08); the goodness of fit index (GFI) was 0.926 (ideal range > 0. 9); the comparative fit index (CFI) = 0.945 (ideal range > 0.93); the incremental fit index (IFI) = 0.946 (ideal range > 0.9); and the parsimony normed fit index (PNFI) = 0.709 (ideal range > 0. 50) (Franke and Byrne, 1995; Steiger, 1990).

### 3.2 Research questions 2

Table 3 shows the findings of the implicit memory experiment.

**Table 3:** Implicit memory experiment results.

Serial number	Statements	J-ratio	Mean response time (ms)	Mask dimension
1	Able to care for family members	76.5%	1,506	F1
2	Good physical and mental health	76.5%	1,608	F2
3	Strong sense of social responsibility	75.0%	1,610	F1
4	Strong cultural literacy	75.0%	1,693	F2
5	Maintains family harmony	75.0%	1,619	F1
6	Strong sense of social responsibility	72.2%	1,503	F1
7	Demonstrates a spirit of hard work and endurance	71.4%	1,533	F3
8	Excellent communication skills	71.4%	1,521	F2
9	Optimistic and positive attitude	71.4%	1,443	F3
10	Demonstrates humility and prudence in handling situations	70.6%	1,515	F3
11	Strong autonomy	68.8%	1,564	F2
12	Kindness and love toward family members	60.0%	1,437	F1
13	High self-confidence and self-esteem	60.0%	1,583	F2

F1 = Collective ideals; F2 = Individual ideals; F3 = Physical and mental qualities.

The mean reaction time for all core statements was 1,549 ms, among which the mean reaction time for the six core words was higher than that for all statements. The “strong cultural literacy” statements had the highest mean response time (1,693 ms), and the “kindness and love toward family members” statements had the lowest (1,437 ms). The statement “able to care for family members” had the highest J-ratio (76.5%), while the statement “high confidence and self-esteem” had the lowest (60.0%). Regarding the three mask dimensions, physical and mental qualities had the shortest mean response time (1,525 ms) and the highest J-ratio (72.5%). Individual ideals had the longest mean reaction time (1,590 ms) and the lowest J-ratio (68.8%). Finally, collective ideals had a mean reaction time of 1,535 ms and a J-ratio (71.7%). The difference between the highest and lowest J-ratios was less than 10%, and no large gaps emerged. The individual J-ratios across the three mask dimensions revealed that 37 participants had the highest collective ideal J-ratios; therefore, they were coded into the collective ideals group. Thirty-one participants had the highest individual ideal J-ratios and were therefore coded into the individual ideal group. Lastly, 32 participants had the highest physical and mental qualities J-ratios and were thus coded into the physical and mental qualities group.

### 3.3 Research questions 3

Table 4 presents the relationships between participant personality masks and parenting behaviors.

**Table 4:** Relationship between personality masks and parenting behaviors.

Mask dimensions	Affectionate–rejection		Autonomous–controlling		Democratic–authoritarian		Intentional indifference–over protective		Consistency–inconsistency		*Cohen’s d*
	*M*	*SD*	*M*	*SD*	*M*	*SD*	*M*	*SD*	*M*	*SD*	
Collective ideals	3.79	0.675	3.78	0.551	3.42	0.727	3.50	0.728	3.95	0.780	0.515
Individual ideals	3.98	0.808	3.95	0.691	3.78	0.555	3.91	0.597	3.49	0.631	0.641
Physical and mental qualities	3.42	0.767	3.46	0.828	3.39	0.549	3.65	0.621	3.60	0.770	0.550
	*F* = 4.677*, df = 2.97 *η*^2^ = 0.088		*F* = 4.057*, df = 2.97 *η*^2^ = 0.077		*F* = 3.938*, df = 2.97 *η*^2^ = 0.075		*F* = 3.491*, df = 2.97 *η*^2^ = 0.067		*F* = 3.752*, df = 2.97 *η*^2^ = 0.072		
df	2.97										
*F*	6.791^**^										
*η* ^2^	0.123										

*p < 0 05, **p < 0 01, ***p < 0.001. M, mean; SD, standard deviation.

Table 4 reveals significant differences in the overall parenting behaviors of participating mothers across the three personality mask dimensions (*F* = 6.791, *df* = 2.97, *p* < 0.01). Significant differences were also observed in the dimensions of parenting attitude across the different personality masks. The personality dimension explained 12.3% of the variance in parenting behavior, which is a moderate effect (*η*^2^ > 0.06 is moderate). The affectionate–rejection attitude showed significant differences across the different personality mask dimensions (*F* = 4.677, *df* = 2.97, *p* < 0.05). The personality dimension explained 8.8% of the variance in the emotional-rejection attitude, which was a small to medium effect. Particularly, the level of affectionate–rejection attitude in the physical and mental qualities dimension (mean [*M*] = 3.42, standard deviation [*SD*] = 0.767) was significantly lower than that in the collective ideals dimension (*M* = 3.79, *SD* = 0.675) (*p* < 0.05). The group with collective ideal had a higher emotional-rejection attitude than the group with physical and mental quality by 0.515 standard deviations, which was considered as a medium effect (d ≈ 0.5 is medium). The autonomous–controlling attitude behavior also showed significant differences across the different personality mask dimensions (*F* = 4.057, *df* = 2.97). The personality dimension explained 7.7% of the variance in autonomous-control attitudes, which is a small to medium effect. For this behavior, the level of the physical and mental qualities dimension (*M* = 3.46, *SD* = 0.828) was significantly lower than that of the individual ideals dimension (*M* = 3.95, *SD* = 0.691, *p* < 0.01). The independent-control attitude of the personal ideal group was 0.641 standard deviation higher than that of the physical and mental quality group, which was a moderately large effect. The democratic–authoritarian attitude showed significant differences in the personality mask dimensions (*F* = 3.938, *df* = 2.97, *p* < 0.05). The persona dimension explains 7.5% of the variance in democratic-authoritarian attitudes, which constitutes a small effect size.with significantly higher levels in individual ideals (*M* = 3.78, *SD* = 0.555) than in collective ideals (*M* = 3.42, *SD* = 0.727, *p* < 0.05) and physical and mental qualities dimension (*M* = 3.39, *SD* = 0.549, *p* < 0.05). The democratic-authority attitude of the individual ideal group was 0.550 standard deviations higher than that of the collective ideal group, which was a moderate effect. The intentional indifference–overprotective attitude showed significant differences across the personality mask dimensions (*F* = 3.491, *df* = 2.97, *p* < 0.05), with significantly higher levels shown for individual ideals (*M* = 3.91, *SD* = 0.597) than for collective ideals (*M* = 3.50, *SD* = 0.728, *p* < 0.01). The persona dimension explains 6.7% of the variance in intentional neglect-overprotection attitudes, which constitutes a small effect size. Considering the consistency–inconsistency attitude, this also showed a significant difference across the three personality mask dimensions (*F* = 3.752, *df* = 2.97, *p* < 0.05), where the level of individual ideals (*M* = 3.49, *SD* = 0.631) was significantly lower than that of collective ideals (=3.95, *SD* = 0.780, *p* < 0.05). The persona dimension explains 7.2% of the variance in consistency-inconsistency attitudes, which constitutes a small effect size.

## 4 Discussion

### 4.1 Research question 1

The statements on the personality masks of young children’s mothers were factor analyzed to form three dimensions—collective ideals, personal ideals, and physical and mental qualities, based on Jacobi’s theory (Jacobi, 1977). To determine the collective ideals’ dimension, we analyzed vocabulary content focused on behaviors toward family members. This reflects mothers’ greater concern for their families. The traditional Chinese concept of family emphasizes the family’s importance as the basic unit of society, and that the closest kinship relationships are among family members (Ho, 1996). For women in particular, family is the most important circumstance to which they belong (Chao, 1994). Therefore, in traditional Chinese culture, women are usually expected to be good wives, mothers, and daughters, and their self-worth is often closely linked to the relationships between family members (Rosenlee, 2006). The influence of this traditional family concept leads to Chinese mothers paying more attention to family life, especially mothers of young children (Chen et al., 2000).

The personal ideals dimension includes cultural literacy, self-confidence, self-esteem, communication ability, and independence. In traditional Chinese culture, mothers play a key role in cultural inheritance (Yiu et al., 2017). Mothers with high self-confidence and self-esteem handle parenting challenges better, fostering positive parenting and children’s wellbeing (Bornstein et al., 2011; Aunola and Nurmi, 2005). Good communication skills enhance understanding and relationships with children, promoting social adjustment (Tamis-LeMonda et al., 2001). Independence enables mothers to address parenting challenges, balance work and family, and improve family harmony (Chuang and Tamis-LeMonda, 2009).

The physical and mental qualities dimension includes mothers’ health, hard-working nature, optimism, modesty, and prudence. A mother’s physical and mental health significantly impacts parenting effectiveness and children’s development (Goodman and Gotlib, 1999; Field, 2010). Hard-working mothers persevere through challenges, providing consistent care (Crnic and Low, 2002; Nomaguchi and Brown, 2011). Optimistic mothers better manage stress, fostering positive parent–child relationships and children’s wellbeing (Scheier et al., 1994; Masten and Reed, 2002). Modesty helps mothers learn from others and build respectful relationships, promoting children’s self-esteem (Lin et al., 2015; Tamis-LeMonda et al., 2001).

### 4.2 Research question 2

The implicit memory experiment results showed small J-ratio gaps between mothers’ collective ideals, individual ideals, and physical and mental qualities’ dimensions, indicating that the personality masks of young children’s mothers maintain a balanced level across the three dimensions. Such balance is essential to a healthy personality mask (Jacobi, 1977). If a mother tends toward collective ideals and neglects individual ideals and physical and mental health, she may over-adapt to society (Kasser and Ryan, 2001). Under these conditions, her main focus is on social needs, which may cause her to neglect self-understanding and her own needs (Deci and Ryan, 2008). This can lead to mothers losing themselves in the process of social change and showing signs of over-integration with others (Huta and Waterman, 2014). If a mother’s personality mask constitutes only her personal ideals and she focuses solely on her own needs, adapting to society becomes more difficult (Chen and Vansteenkiste, 2020). Mothers of this type become self-centered because they live in their unrealistic world, without a real mask. Furthermore, a mother who neglects her own physical and mental health in favor of collective and individual ideals may develop neurosis and psychosomatic illness (Chen and Vansteenkiste, 2020).

A comparison of the questionnaire and experimental results revealed that, at the questionnaire level, the three dimensions were ranked as collective ideals, personal ideals, and physical and mental qualities. However, the rankings in the implicit experiment were in the order of physical and mental qualities, collective ideals, and personal ideals.

Research shows people prioritize collective ideals consciously due to their link to social identity and belonging, seeking group consistency for acceptance (Tajfel and Turner, 1986). Personal ideals focus on achievement and self-actualization, fulfilling inner needs and self-worth (Huta and Waterman, 2014). Physical and mental qualities, crucial for quality of life, are secondary consciously but prioritized unconsciously due to their role in survival and reproduction (Bargh and Morsella, 2008).

Next, collective ideals relate to the human instinct to live in groups and socialize (Baumeister and Leary, 1995). People unconsciously focus on collective ideals to maintain harmonious social relationships and internal stability (Brewer, 1999). While personal ideals are key to growth and development, they may be relatively minor factors at the unconscious level (Deci and Ryan, 2000).

### 4.3 Research question 3

Parenting behaviors differed significantly across personality mask dimensions. Mothers focused on collective ideals showed more love and less neglect or punishment compared to those emphasizing physical and mental qualities (Baumrind, 1991). Parents with personal ideals prioritized autonomy and self-regulation (Ryan and Deci, 2000, 2017; Darling and Steinberg, 1993). In contrast, those focused on physical and mental qualities emphasized external success, often neglecting children’s inner needs, leading to controlling parenting (Twenge and Campbell, 2009; Barber, 2002).

An autonomous–controlling parental attitude revealed differences in personality mask dimensions, with physical and mental qualities lower than personal ideals. Mothers focused on personal ideals prioritize children’s autonomy, adopting self-disciplined parenting that grants freedom and responsibility (Ryan and Deci, 2000; Deci and Ryan, 2008). In contrast, mothers emphasizing physical and mental qualities prioritize health, using controlling parenting to ensure healthy habits and emotional regulation (Darling and Steinberg, 1993; Barber et al., 1994; Baumrind, 1991; Chen et al., 2000).

A democratic–authoritarian parental attitude revealed higher personal ideals than collective ideals and physical/mental qualities. Mothers with personal ideals adopt democratic parenting, prioritizing children’s autonomy, emotional needs, and self-expression, fostering self-esteem, confidence, and social adjustment (Deci and Ryan, 2000; Ryan and Deci, 2000). In contrast, mothers with collective ideals or physical/mental qualities emphasize conformity, obedience, and controlling behaviors (Chao, 1994; Darling and Steinberg, 1993; Kim et al., 2013; Maccoby and Martin, 1983).

The intentional indifference–overprotective attitude showed higher individual ideals than collective ideals. Mothers with individual ideals prioritize children’s independence, using supportive guidance (Kim and Rohner, 2002; Grolnick and Ryan, 1989), while collective ideals mothers emphasize compliance, often using controlling or punitive methods, leading to overprotective behaviors (Chao, 1994; Grolnick and Ryan, 1989). For consistency–inconsistency attitudes, collective ideals mothers exhibit more consistent parenting, aligning with social norms (Triandis, 1995b), whereas individual ideals mothers use flexible practices to meet children’s needs (Triandis, 1995a).

## 5 Implications for research and practice

This study identifies three components of mothers’ personality masks: collective ideals, individual ideals, and physical and mental qualities, aligning with Jacobi’s theory. Collective and individual ideals form in homeschooling, requiring a focus on individuality, reduced pressure, and improved social adaptability (Soenens et al., 2007; Zhang et al., 2020). Physical and mental health significantly impacts mothers’ personality and children’s development, emphasizing the need for exercise, rest, and stress reduction (Giallo et al., 2014; Bennett et al., 2016). Maintaining routines, diet, and resilience is crucial for mothers’ wellbeing (Seligman, 2011).

Mothers with collective ideals should balance group interests with individual needs, respecting each child’s uniqueness (Darling and Steinberg, 1993). Ideal mothers must avoid excessive control, respect autonomy, and provide freedom (Baumrind, 1991). Democratic mothers should balance autonomy with guidance, respecting opinions while setting limits (Maccoby and Martin, 1983). Indifferent mothers must meet children’s basic and emotional needs with attention and affection (Grolnick et al., 2013). Consistent mothers should consider individual differences and create personalized parenting plans (Bornstein and Cheah, 2006).

There is also a need to enhance the education and support of mothers of young children to improve their parenting skills and confidence (Bornstein, 2019). For example, they can be provided with the necessary support and guidance to help them cope better with the stresses and challenges of parenting through home visits, parenting courses, and psychological counseling (Morawska and Sanders, 2009).

## 6 Limitations and future study prospects

First, the sample was only taken from 233 mothers in the Kindergarten of Region Z. Although this ensured internal validity, the high cultural homogeneity limited the external generalizability of the conclusions, especially in the context of China’s diverse regional cultures;

Secondly, Although the implicit memory experimental paradigm in this study is based on the method proposed by Lee et al. (2017), the artificial experimental environment lacks ecological validity—and thus fails to fully capture the unconscious processes in real-world parenting scenarios.

Third, in the scale development process, items were screened through expert rating and factor analysis. However, item response theory (IRT) was not applied to verify item discrimination and difficulty—which may compromise measurement precision.

Fourth, the cross section has certain limitations, there may be two-way influence or third-party variables interference;

Fifth, this study did not include a control group. We plan to incorporate women without specific parenting roles or fathers in future research to more clearly distinguish whether the current pattern is unique to the maternal role or a common trait across broader groups.

Finally, statistical methods such as ANOVA confirmed between-group differences, they failed to control for the inflation of Type I errors caused by multiple comparisons—nor did they employ multivariate analysis to more comprehensively examine the relationships between variables. Additionally, the study revealed that between-group differences are statistically significant yet have small effect sizes; this outcome may be attributed to the cultural homogeneity of the sample and the characteristics of the measurement instruments.

To address these limitations, future work of this study will expand the representativeness of the sample, incorporate behavioral observation methods, consider including experimental control groups or conducting cross-validation, optimize measurement instruments, introduce a longitudinal design, and employ more advanced statistical models. Additionally, future research may adopt analytical frameworks with greater explanatory power—such as structural equation modeling (SEM)—to further validate and refine the findings of this study.

## Data Availability

The original contributions presented in the study are included in the article/supplementary material, further inquiries can be directed to the corresponding author.
